# Doctors as moral pioneers: Negotiated boundaries of assisted conception in Colombia

**DOI:** 10.1111/1467-9566.12979

**Published:** 2019-07-21

**Authors:** Malissa Kay Shaw

**Affiliations:** ^1^ The Graduate Institute of Humanities in Medicine Taipei Medical University Taipei Taiwan

**Keywords:** assisted reproductive technologies, moral pioneers, improvised ethics, bioethics, biotechnologies, private health care, Colombia

## Abstract

New biotechnologies such as assisted conception are socially embedded artefacts that raise context‐specific ethical, moral and social anxieties. In contexts where the regulations of these profitable developments are limited or ambiguous, and competition between private facilities is high, individual doctors become morally and socially responsible for determining the parameters of administering such therapies. Ethnographic research at two private fertility centres in Colombia reveals that doctors do not determine boundaries based on monetary gain but rather personal morals, social norms and professional obligations. Medical professionals hold diverse perceptions of assisted conception, and often struggle to make decisions regarding who should access such therapies, who are ideal gamete donors and the fate of extra embryos. The complexity of these perceptions applied in a context of limited regulation and the competition of private medicine impacts the praxis of assisted conception. As doctors determine the boundaries of their practice they not only create variation between clinical practices, but also make moral decisions regarding who should be parents, how families should be formed and the significance of embryos. Thus, in navigating their everyday practices, doctors also shape the social world.

## Introduction

In her seminal work, Rapp (1999; see also Rapp 1988, 2011) proposes the term ‘moral pioneers’ for women who traverse uncharted territory as they engage with new reproductive technologies, specifically amniocentesis, and make decisions about the fate of their pregnancy based on available, long‐standing resources. These women used their own desires, notions of family and disability and perceptions of the technology in deciding whether to terminate a pregnancy that technology determined to be affected. Rapp's (1988: 110) findings point ‘to a cultural diversity of both problems and solutions’ arising from these technologies. The range of possibilities that new biotechnologies present creates hope ‘for some and profound unease for others’ (Melhuus 2012: 109). This hope and unease surpasses patients alone, also affecting medical professionals, especially in settings where limited legislation regulates the use of biotechnologies. There is now a substantial body of research in medical anthropology and sociology that examines patients’ moral decision‐making around reproductive technologies. While research on how doctors grapple with ethical and moral dilemmas that accompany the globalisation of these technologies is growing (e.g. Gammeltoft 2014, Hörbst 2012, Roberts 2012, Simpson 2004), more research is needed. In this article, therefore, I explore how medical professionals negotiated the ethical, moral and social boundaries of assisted reproductive technologies (ARTs) in the context of limited regulation and the ‘big business’ of assisted conception (Ryan 2009: 805) in Colombia. Following the work of Livingston (2012), I propose that medical professionals in this context must engage in ‘improvised ethics’ as they improvise treatment practices and ethical boundaries which have considerable implications for both patients and practitioners themselves.

## Local moralities of assisted conception

ARTs are culturally embedded artefacts formed by human and non‐human factors, including the technical aspects of ARTs and the ‘economic, political, cultural and moral environs in which they unfold’ (Inhorn and Birenbaum‐Carmeli 2008: 178). Given their context specificity, ARTs have drawn diverse reactions as they have entered new locales, such as opposition from the Catholic Church due to the manipulation of embryos and doctors playing the role of God (Roberts 2006: 507), or their perceived reinforcement of Buddhist principles as they aim to relieve the distress and stigma of infertile couples (Simpson 2013: S88). As their acceptance or rejection depends on ARTs corresponding with ‘deeply embedded religious and ethical traditions’, exploring these technologies reveals local moralities and the cultural construction of social values (Inhorn 2015: 22; see also Hedgecoe 2004: 126).

In certain contexts accepting assisted conception has involved creatively reinterpreting biological and social processes. For instance, doctors created the ‘pre‐embryo’ to differentiate between the implanted embryo and the entity that exists between in‐vitro fertilisation (IVF) and implantation and to justify the manipulation of (pre‐)embryos and IVF within the moral terms of oppositional groups (Raymond 1988: 281). Notions of kinship have also been reconstructed in particular contexts, such as Ecuadorian couples refuting the significance of gametes, but embracing the connection made through the exchange of blood in‐utero in order to accept egg donation as a socially acceptable means of conception (Roberts 2008). With the steady development of new ART techniques, such as combining therapies (e.g. IVF with PGD) or new combinations of people seeking treatment (e.g. two homosexual men employing a surrogate mother), which challenge dominant ethical, legal and religious ideologies, we will continue to encounter new moral and social dilemmas in specific contexts (Williams *et al*. 2005).

Legal procedures have been put in place in certain countries to regulate medical practice and protect patients. Laws and their enactment ‘are significant because they … reflect dominant social concerns and values’ (Melhuus 2012: 3). Singer's (2017) research on newly legalised abortion in Mexico City, for instance, demonstrates that this outward expansion of reproductive rights essentially reiterated long‐standing state and social values around reproductive responsibility. Undergoing a state‐funded abortion through Mexico City's public abortion program is accompanied by a lesson in responsible sexual practices intended to prevent (another) unwanted pregnancy. However, medical practitioners do not always justify their practice within the framework of legal (or social) ideology. In the context of late term abortions in Vietnam, Gammeltoft (2014: 110) shows that doctors felt it was their responsibility to determine the ambiguous line between mild and severe foetal anomalies in order to provide women and families with healthy children – not to meet ‘state‐set demographic goals’. Despite resulting in the same medical practice, these viewpoints were based on different ideologies of responsibility.

When legislation is not established or enforced and practices are not standardised, doctors become responsible for navigating this unexplored terrains and negotiating their position on certain techniques as they determine which practices correspond with their own moral worlds (Kleinman 1992). In such contexts, doctors’ perceptions and practice may be directed by guidelines set by international professional societies and bioethical principles. Such principles, however, may not correspond with cultural norms or apply in practice. Simpson (2013: S87) claims that tension may exist between the ‘vernacular’ of local ‘meaning, morality and kinship … and the powerfully naturalising discourse of law, regulation and bioethics in which’ ARTs are ensconced. In Sri Lanka, for example, the traditional kinship practice of polyandry supports intra‐familial sperm donation, meaning that ‘passing sperm between known persons’ is an appropriate way to conceive using ARTs (Simpson 2004: 234; see also Simpson 2013). Despite corresponding with local understandings of assisted conception, the practice of known gamete donation contradicts the medical guidelines adopted from abroad, which stress anonymity and the doctor's role as mediator between donor and recipient. These opposing perspectives create a divide between ‘local ideas of substance, exchange and connection’ and what some doctors perceive as universal bioethical standards (Simpson 2013,: S88‐S91). Social scientists have long criticised bioethics for being acultural and rooted in philosophical theory rather than adopting a position that sees medical dilemmas and their bioethical resolutions as context‐specific, continually evolving and needing to be explored through empirical evidence (Hedgecoe 2004, Marshall 1992). When the latter is taken into consideration, we see a different picture. As Silva and Machado's (2011) research from Portugal demonstrates, although fertility specialists may emphasise the universality of medical knowledge and technical procedures of reproductive technologies that inform their clinical judgment over that of local regulations, this does not mean that they deny the impact that local values and norms have on their practice. Simpson (2013: S92) suggests that the power of biomedicine will undermine the meaning of the vernacular, but ultimately biomedical practice and ‘universal’ bioethics are inevitably entangled in local beliefs and norms.

All this is complicated by the financial demands and incentives of private medicine. Competing in the globalising enterprise of reproductive technologies may infringe on a presumed altruistic character and ethical commitment of medical professionals. As I will explore in this article, given the lack of legal regulations in Colombia and the monetary demands of managing a high‐tech fertility clinic, doctors may feel pressed to make treatment decisions based on financial gain. In analysing doctors’ narratives, however, I show that various factors shaped doctors’ use of certain ART techniques. Treatment boundaries were not negotiated based on monetary gain but rather personal morals, social norms and professional obligations to their patients. Medical professionals held different understandings of their acceptance of assisted conception and the individuals who should access such therapies, and at times struggled with certain treatment decisions.

At the heart of ARTs and the ‘sociocultural cum political processes’ that surround them ‘are not only problems of conception but also … meanings of conception’ (Melhuus 2012: 2). In setting boundaries for their practice doctors also shaped boundaries about ideal parents, family formations and the significance of biogenetic materials. Considering this, I employ the theory of moral pioneers, to highlight how individual moral principles can (re)shape social constructs creating both opportunities and challenges for those engaging with new biotechnologies.

## Research Methods

The findings discussed in this article were part of a larger study that explored couples’ and medical staff's experiences and perceptions of ARTs in Colombia (Shaw 2016, 2018). In 2012 and 2013, I carried out 10 months of ethnographic research at two high‐tech fertility centres in Bogota. There I observed the everyday workings of the facilities, including doctor–patient consultations, medical examinations, ART procedures, reproductive surgeries, laboratory proceedings and interactions between medical personnel. I also conducted interviews with clinic staff (four fertility specialists, an urologist, five embryologists, four nurses, four administrators, a cleaner, a financial investor for a clinic and an anaesthesiologist), a family practice lawyer with experience working with fertility clinics, and 74 women, some of who were accompanied by their partner. These women or couples came from all parts of Colombia, and the majority were middle‐upper or upper class professionals who had the financial resources to access ARTs, which are only available at private medical centres.

Interviews were conducted in the clinic behind closed doors, and I sought verbal consent from participants before each interview. I conducted most interviews in Spanish, although some informants insisted we speak in English, as they wanted to practice their English or were bilingual. The interviews with medical staff were audio recorded, and later I transcribed and translated them to English. Two medical staff chose not to be audio recorded, thus the details of these interviews were noted by hand during and immediately after the interviews. Due to a number of patients’ evident unease at the beginning of the study, I did not audio record interviews with patients. Instead, I took detailed notes both during and directly following the interview, as recommended by Inhorn (2004: 2099). Interviews with medical staff and the lawyer were semi‐structured and partly specific to the informant's specialisation, while interviews with patients were unstructured and in‐depth. I also recorded informal conversations that took place throughout the ethnography in my fieldwork diary.

Using NVivo Data Analysis Software, I compiled and coded all the data line by line using themes that I identified in the dataset and those from the literature. This permitted recurring themes, and the diverse opinions and experiences of informants to be identified. I have changed all names and identifying characteristics of participants and the medical facilities. The University of Edinburgh's Ethical Review Board approved this study.

## Assisted conception in Colombia

Article 42 of Colombia's 1991 Constitution states that ‘children born from matrimony or outside it, adopted or conceived naturally or with scientific assistance, have equal rights and duties’. According to a family law attorney I interviewed, this Article approves the use of ARTs and protects children conceived through such techniques, as well as those who administer and make use of these treatments. Notwithstanding this constitutional article, other ART regulations are minimal.

Congressional decree 1546 of 1998 requires that biomedical facilities offering ARTs and their laboratories be registered and regulated and that fertility experts undergo accreditation. It also provides guidelines for necessary diagnostic examinations for ARTs to be considered a viable treatment and for the registration of donors. The only regulation specifically restricting treatment pertains to the manipulation of embryos and the practice of ‘designer babies’. The most prescriptive regulations have been adopted from organ donation legislation and concern the use of donor gametes: all donors must be at least 18‐years‐old, screened for infectious diseases, and undergo physical, genetic, and psychological examinations. Gamete banks should be anonymous, but all donors must be registered with an identification number. According to regulations governing the donation of all biological materials, gamete donation should be voluntary and altruistic.

In 2003 and 2004, the limitations of these laws were brought to the attention of Congress by two groups of Senators who were influenced by prolife Catholic lobbyists. This led to an extensive debate over the regulation of ARTs. The groups put forth new bills to further restrict permitted techniques, including restrictions on who can access the procedures (i.e. heterosexual married couples, single women, etc.) and specifications for leftover biogenetic materials. The proposed bills were not passed into law. Again in 2015, assisted reproduction came to the forefront of political deliberations, but this time in regard to recognising infertility as a disease and requiring treatment (including ARTs) to be covered by the *Plan de Beneficios* (Benefits Plan, also known as the Mandatory Health Plan). The proposed bill (*Proyecto de Ley 082 de 2015*) was ratified by the *Cámara de Representantes* (House of Representatives) in 2016, and then sent to the *Senado* (Senate) where it was ratified in June 2017. However, during the following month the Colombian President, Juan Manuel Santos, vetoed the bill, claiming that the coverage of ARTs by the *Plan de Beneficios* would be financially unsustainable (El Tiempo 2017). Santos’ explanation echoed the government's previous stance on ARTs: Decree 806 of April 30, 1998, Article 10 states, ‘the Mandatory Health Plan is not obliged to contribute to the diagnosis, treatment, or rehabilitation of sicknesses that are considered cosmetic, aesthetic, or sumptuary, or that are a complication resulting from these treatments or processes’ (author's translation). This decree classifies ARTs as unnecessary and extravagant expenditures that the government cannot afford. Whether other provisions proposed in the 2015 bill will be passed in the future – such as the creation of a national registry and the increased monitoring and control of ART centres – remains unknown.

Despite congressional attempts to further regulate ARTs, laws remain limited creating an uncertain legal context for infertility specialists. Hörbst (2012: 49) described a similar ‘ambivalent situation’ in Mali where the ‘legal lacuna allows physicians a professional liberty for practicing all variants of ARTs according to what they consider’ to be necessary for successful treatment, but this lack of legal clarity also ‘disrupts their private investments as future regulations might prohibit some treatments or variants (for which they already have invested)’. The fertility specialists who participated in this study were relieved that none of the bills proposed in 2003‐2004 passed into law. Dr. Mabel, the lead specialist and owner of a fertility clinic, explained that given the influential Catholic lobby it is better that infertility treatment remains unregulated to prevent his practice from being restricted. Dr. Herrera, a fertility specialist and co‐owner of a clinic, echoed this sentiment:

If we have a very conservative government, it is possible that we will not be able to do the things we want to do … often unnecessary laws are created but important factors are passed over. Because of lack of knowledge, people … who regulate these practices … do not understand our work.

The lack of legislation makes Colombian doctors the primary regulators of the treatment options they are willing to offer. This is even more pronounced because treatments take place in the private sector where the limited regulation that does exist is rarely enforced (Roberts 2006: 521). The doctors who participated in this study were members of international medical bodies, such as the Latin American Network of Assisted Reproduction (REDLARA), an educational and scientific institute that offers guidelines to direct practice, but these guidelines are neither binding nor are they always applicable to real world situations. Furthermore, as Simpson (2013: S87) explains the vernacular does not always correspond with the ‘push towards globalisation, standardisation and the universalisation evident in the ethics of ARTs’. The lack of clear practice regulations has created treatment variation across and even within clinics, based on the treatments doctors are *willing* to perform, and the clinic's resources and expertise that influence the services they *can* offer.

## Medical experts or entrepreneurs?

Medical practitioners were largely responsible for the introduction of ARTs to Colombia. According to Dr. Mabel, a workshop in Barranquilla in 1982 was the initiation point: ‘Everyone who was interested in ARTs was there’. Following this workshop, the first clinic offering ARTs, *Centro Colombiano de Fertilidad y Esterilidad* (CECOLFES), was founded in Bogota by four ART specialists, and shortly thereafter another clinic, *Genes*, opened in Medellin. CECOLFES’ procedures resulted in the first clinical IVF pregnancies in Colombia, followed by the birth of the first IVF baby in the region, Diana Carolina Mendez, born on 10 January 1985.

In 2018, 13 Colombian clinics were registered with REDLARA. However, an interview in August 2014 with a representative at Merck – the pharmaceutical company that supplies the majority of hormonal drugs used in ART cycles in Colombia – suggested that there were approximately 50 clinics offering ARTs in Colombia. Although this industry has expanded throughout the country, access to fertility treatment is constrained due to various factors, foremost their prohibitive costs and lack of information, which has been perpetuated by the government's disregard for infertility (Shaw 2016; also see Inhorn 2003).

Under current legislation, neither the Mandatory Health Plan nor private medical insurance cover infertility treatment, which effectively limits access to those who can pay. Treatment costs, excluding medications, range between US$900 for artificial insemination and US$5400 for intracytoplasmic sperm injection with donor eggs.^1^,^2^ These prices are unaffordable for the majority of Colombians who earn a legal monthly minimum wage of US$262 (Cobb 2016). Through the adoption of the *tutela* (or writ of protection) in the 1991 Constitution, individuals who wish to access ARTs may claim their sexual and reproductive rights and right to health care, as guaranteed by the Constitution, are being violated through their denied access to government funded infertility treatment. According to Dr. Herrera, those who have invoked these rights through *tutelas* have generally had the costs of ARTs and/or of the accompanying medications covered by the Ministry of Health. However, few cases have been brought to the attention of the Courts.

High treatment costs are (partly) perpetuated by the substantial financial and educational investments required by medical practitioners and others to procure the necessary clinical space, technologies and trained personnel to run an ART clinic (Gerrits 2012: 4). Lacking the essential start‐up funds, many Bogotano clinics initially used the resources of major hospitals (clinical space, microscopes, incubators, etc.), and were affiliated with, but not run or financed by, these large institutions. As clinics have increased their financial capital through consistent treatment demand many have become autonomous enterprises. Other practitioners acquired the necessary funds through their own private investments or that of third parties.

Infertility specialists also incur the expenses of learning the practice and developing a team of highly skilled medical professionals. Until 2017, no medical training for ARTs was available in Colombia. Furthermore, according to Dr. Samuel, an embryologist, medical students and teaching faculty at Colombian institutions lack awareness about the study of clinical embryology and assisted reproduction, limiting the number of professionals who specialise in these fields. Similar to Roberts’ (2006) observations in Ecuador, most aspiring doctors and embryologists receive training in other Latin American countries, or at European or North American institutions. This international training lends prestige and a ‘modern’ valence to infertility care.

The combination of the doctors’ entrepreneurial character and their large financial investments fuels competition and restricts cooperation between doctors and clinics. Dr. Herrera explained that she does not collaborate with other clinics because it is private medicine: ‘We are all jealous of the other. There is collaboration at conferences and other academic events, but that is the extent of it’. Dr. Samuel echoed this, ‘Clinics do not interact with other clinics … there is a lack of trust. It is competition’.

This competition inhibits collaboration between doctors working at different facilities *and* perpetuates the limited awareness about ARTs in Colombia, which constitutes a barrier to treatment access and reduces the potential for collaboration with other professionals working in the field of reproductive health (e.g. psychologists, bioethicists, policymakers). As Dr. Herrera explained, this effectively eliminates an avenue for deliberating difficult situations that arise during practice:

I would like to discuss [certain issues] with other people, with psychologists, with sociologists to decide something … the interrelation of a committee would make it much easier to propose certain things, because it would not only be a doctor [making the decision], but here there is not a committee.

The lack of collaboration and institutional proceedings to guide ART practices largely makes individual doctors responsible for determining treatment guidelines. Kerr (2004: 82) explains, ‘When professionals give clients choices they also give them responsibilities for those choices, which can be burdens in their own right. In many ways, this version of choice privatises responsibilities …’ Kerr was referring to medical professionals allowing patients to make genetic screening related decisions during pregnancy. This same logic, however, applies when doctors are responsible for making medical, as well as social and moral, decisions in the context of limited legal regulations, inadequate collaborative support from other professionals, and the financial responsibility that accompanies managing a clinic in a private medical system. As I will show, even though doctors took an entrepreneurial approach to their practice, they were not merely concerned with generating profits. Rather, they often based decisions on their own moral and ethical reasoning about what treatments they were willing and able to offer.

## Negotiating practice boundaries

It is obvious that we have to work with a lot of things that … are not only medical things. We have to think in ethics. We have to think in social questions. (Dr. Herrera)

Medical professionals in this study were confronted with a variety of treatment deliberations such as determining who should undergo ARTs and when treatment should be denied or terminated, who can be gamete donors and the ideal fate of extra embryos. These decisions are entwined with local moral and social understandings that influence medical practice, and question the notion of acultural and universal bioethics (e.g. Hedgecoe 2004, Marshall 1992, Simpson 2004). Rather, doctors justified their medical practices based on personal morals, social norms and professional obligations to their patients.

### Denying and foregoing treatment

At the clinics where the study was conducted, patients did not have to meet a particular profile to receive treatment (e.g. marital status, sexual orientation, age). Ultimately, if they could pay, they could undergo treatment. However, patients presenting specific characteristics raised concern among the fertility experts that at times resulted in treatment delays. Dr. Herrera, for instance, discussed her concern about the age of female patients:

It is very difficult … actually we attend women older than 45‐years‐old, sometimes. We have reviewed this to consider a [age] limit, and finally it is an arbitrary limit, because one cannot say what the [medical] risk of pregnancy is with a woman of 45.5 years. Thus for us the limit is 45 but we have done treatment with women who are older because the risk was not high enough to justify saying we cannot. But usually it is 45 … but there are many things in these cases that do not only involve the doctor. It is one thing to think about risks to health and another is … the quality of life of an adolescent with a mother who is 60 years old or more. These are questions that do not have an answer.

Although Dr. Herrera could deny a 45‐year‐old woman treatment based on medical grounds, the situation was more complicated when couples presented relationship complications.

There are couples that one can perceive are not very functional, but at the moment that you need to reject someone, this is complicated, because you have to think that they do not have a psychological problem but they are very dysfunctional. So in this case, we prefer to work more with the psychologist before the couple seeks treatment.

Similarly, Dr. Mabel recommended that Mariela, a 34‐year‐old biologist, delayed treatment due to her weight, ‘He told me I need to be healthy, lose some weight, before getting pregnant to avoid weight related complications during pregnancy’. Mariela appreciated the doctor's concern for her overall health and that he did not push her to initiate treatment immediately, but she was surprised that he recommended she delay the treatment cycle.

A couple's relationship status, a woman's age or her weight, and psychological conditions were all reasons that fertility specialists delayed or prevented patients from undergoing treatment at their clinic. One may argue that these issues are (directly or indirectly) medically (as well as socially) related to a couple's ability and fitness to have a child. However, medical professionals are not necessarily consulted about these issues when a couple tries to conceive without medical intervention. As Melhuus (2012: 16) explains, in regulating ARTs, either through legislation or medical authority, the focus of reproduction shifts from individual choice to social acceptability. By embracing their understanding of socially acceptable parenthood, these fertility specialists were taking the responsibility to further regulate who should access treatment, who should have the potential to become parents. Doctors made such decisions to delay treatment knowing that doing so would reduce their revenue in the interim, and potentially in the long term as patients may go elsewhere or speak negatively about the centre to potential patients (Shaw 2016: 119).

I should note that medical personnel who were not the lead fertility specialists felt that they could not intervene in the social lives or discuss the psychological well‐being of patients. Dr. Diaz, a fertility specialist trainee, explained that he cannot tell a ‘couple they should not go through fertility treatment because … they do not have a good enough relationship, they fight too much, or they will not have the time to raise the child’. He said he cannot ‘say those things or play the role of an intermediary’ because he would lose patients, but also because that is not the role he is seen to play as a doctor.

Dr. Samuel also felt that his role was not to judge patients’ suitability for treatment, as he expressed while discussing his reservations about an 80‐year‐old man with eight children seeking fertility assistance with a 40‐year‐old woman. He stated,

I cannot tell them no, because it is my job. My job is to help them have a child and if he is here, then he wants a child … so I try to help them … I believe there is a higher energy here called God. I think he ends up helping or choosing as well. Some people call it nature … but nature rules, nature is the one that makes the decision.

Both doctors felt that their responsibilities did not include denying patients access to treatment, even if they harboured judgements about couples’ reproductive choices. Rather they were supposed to assist in facilitating conception. Dr. Samuel's religious convictions showed he believed a higher power would ultimately choose whether a couple became pregnant (see Roberts 2006), therefore, he did not need to worry about what for some may have been a morally difficult decision. Moreover, in the case of Dr. Diaz the lead specialist oversaw his practice. Ultimately, then, such decisions would still be made, but the responsibility was transferred to someone else.

Complications arising once treatment was already underway also required medical staff to recommend abandoning the cycle. For instance, when none of the embryos were of high enough quality to be transfer, Dr. Mabel explained,

At some clinics they may transfer the embryos, but for what reason? Just to make the woman realise in 10 to 15 days that she is not pregnant … giving the patient hope while she waits for the pregnancy results? [That is why] I chose not to transfer any of the embryos because emotionally it is better for the patient. It was a hard decision to make but one I had to [make].

A similar decision was made at an earlier treatment stage when a woman's aspirated eggs were too immature for fertilisation. Showing clear disappointment, a senior embryologist, Dr. Catalina, explained,

Adriana's eggs are at the first [developmental] stage. It may be possible to fertilise the eggs, and they may develop into embryos, but they will not implant. So there is no point in fertilising the eggs and giving Adriana false hope that the embryos will implant.

Although in both cases treatment cycles could have continued until the final stage, the medical staff knew they would result in failure, making it best to end the cycles early. Doing so, resulted in revenue loss for the clinic; however, it was intended to reduce the couples’ suffering. Dr. Mabel explained,

Sometimes I have to make hard decisions, but I want to do what is best for the patient in all regards. This process is emotionally and psychologically difficult for the patients. Thus I need to do what is best for them in all these respects: medically, emotionally and psychologically.

Despite being private clinics with hefty financial investments, medical staff did not base their decisions merely on generating profits, but rather the best interests of the patient or their interpretation of socially acceptable parenthood. In deciphering these parameters of their practice, doctors had to creatively manoeuver in unchartered ethical terrain without a collaborative community or regulations to guide their decisions.

### Donor conception practices

Although there is detailed legislation about the age, psychological and biological health, and medical tests that anonymous gamete donors must undergo, no regulations address the number of couples to which a donor can donate or how many of those donations result in pregnancy. By law, donation should be altruistic, aligning with idealised notions of ‘gift giving’ and ‘solidarity’ between donor and recipient (Bestard 2004). In reality, however, ova donors receive roughly US$250 per donation, a payment that on paper appears to cover lost wages and transportation costs but is actually seen by medical staff, donors, and patients as payment for the ‘donation’. Moreover, some fertility specialists permit gamete donation from known donors, which creates treatment variation across (and within) clinics and encourages patients to search for clinics that will provide the service they desire.

Due to the lack of regulations, some clinics have adopted their own guidelines to further standardise treatments. Dr. Herrera explained,

It is important that there is specific control … real control over how many donations a donor makes, and of these [donations] how many are successful [pregnancies]. These are things that we regulate, because it is important, but there is no government control.

Dr. Herrera also prohibited known donor selection. Her partner, however, permitted the practice. Their opposing positions on known gamete donation created variation in the protocols offered in their clinic. All couples requiring a donor and attending Dr. Herrera's practice had to use a donor from the anonymised bank or consult a different doctor such as her partner. Here, having a clear policy against known donor conception prevented Dr. Herrera from being confronted with individual situations that may pose moral or ethical dilemmas.

Alternatively, despite Dr. Mabel's preference for anonymous donation to prevent any social repercussions, paternity or maternity issues, or the donor requesting compensation later on, he permitted patients that requested to use a known donor to do so because, ‘I can do it’, and ‘that [is] their choice’. His desire to accommodate patients' requests in regard to donor selection did raise concerns in certain instances, such as when Jessica, a 42‐year‐old secretary, expressed an interest in using her 20‐year‐old daughter's eggs with her new husband's sperm during a discussion with a nurse and myself. Dr. Mabel was astounded when he became aware of Jessica's intentions: ‘I cannot do that … the child, he would be her grandchild but she would be his mother … it is too complicated … I cannot do it’. Although I had previously observed Dr. Mabel permit sisters to donate eggs to each other, he found this suggested family formation with its generational overlaps to be unfathomable. Unlike the doctors in Simpson's (2004, 2013) example cited above – who rejected intra‐familial sperm donation based on prepackaged standards from abroad – Dr. Mabel based his rejection of Jessica's desired family formation on his own moral volition and understandings of socially acceptable family formations.

### Cryopreserving extra embryos

I found it difficult to directly engage patients in discussions about the significance of embryos. Medical staff repeatedly explained it was because ‘most couples do not want to think about the significance of the beginning of life, what embryos mean … they just want a baby … and thinking about these issues only complicates the situation’. This reluctance posed a barrier to couples deciding what to do with embryos left over after an IVF cycle. Extra embryos can be discarded or cryopreserved for a future cycle. Some clinics also allow extra embryos to be donated to science or another couple.

One clinic in this study allowed extra embryos to be donated to other couples, a practice that Dr. Catalina greatly supported because of an increase in single women inquiring about assisted conception. Couples, however, were reluctant to donate what they saw as a product of *their* bodies, *their* genetic material, ideas that were connected to *their* future child. Dr. Herrera, who did not permit embryo donation, reiterated this notion stating that extra embryos ‘are embryos of [those] patients’. For Dr. Herrera, creating an embryo from separately donated egg and sperm was an acceptable means to create a child. However, transferring another couple*'s* embryo to a woman in the attempt to give her (and her partner) a child was problematic, as that embryo was intended to be part of that couple's family, and therefore was already enveloped in a kin network.

Couples did not see donating extra embryos to science to be a viable option, either, and cryopreserving extra embryos incurs an additional cost. Thus most couples ultimately choose to discard extra embryos. Although the medical professionals acknowledged that the decision is ultimately the patients’, discarding embryos was seen as problematic for both practical and moral reasons. Dr. Ortero, a fertility specialist, explained that it is illogical not to cryopreserve extra embryos: ‘If we say [for example] that treatment is only 50 per cent successful, than there is a 50 per cent chance [the couple] could use those embryos in the next cycle’. What was more problematic for some medical staff, however, was that not cryopreserving extra embryos means discarding them and in cases of severe infertility greatly reducing the likelihood for a successful cycle in the future.

Discarding embryos was clearly a morally trying task for some embryologists. On separate occasions, all three embryologists at one clinic emphatically explained how discarded embryos are not tossed in the bin, but left in the incubator to dry up gradually. Dr. Samuel explained,

[We] let them go slowly, in a non‐painful way, as if they could feel, but they do not have a central nervous system so I know they are not feeling pain, but they are cells, they are living things, they are alive. So we give them a little dignity as far as we are concerned, letting them go in the incubator.

Despite their scientific knowledge that embryos are not sentient, moral notions of embryos being the initiation of life and therefore a potential living being affords them the dignity to perish in what embryologists perceived to be a humane manner. The embryologists also justified the destruction of embryos based on their probability to develop into human beings. Dr. Samuel emphasised that only 30 per cent of embryos have the potential to develop into human beings, ‘and if the patient gets pregnant then the probability of all the other embryos becoming human beings is actually lower’.

Discarding embryos also raised concerns about the likelihood of future treatment success, such as in the paraphrased situation described by an administrator:

A man underwent a testicular biopsy to encounter semen to fertilise his wife's eggs. An embryologist was concerned about not freezing the subsequent extra embryos because if treatment would fail it would be highly unlikely for more sperm to be encountered. Thus, the embryologist proposed cryopreserving any remaining embryos even if the couple refused to pay.

Although cryopreserving extra embryos without the couple's consent would be ethically questionable, the embryologist was expressing her concern for the couple's future potential to become parents, irrespective of the financial burden it may place on the clinic.

Regardless of the anxieties discarding embryos pose for some medical staff, cryopreserving extra embryos is also problematic. Staff at one clinic complained about the lack of storage for all the embryos they had cryopreserved and the defaulted on payments for the stored embryos. Patients who were contacted about this predicament often said they did not want their embryos destroyed or donated, that the clinic should do nothing. Akin to concerns discussed by doctors in Mexico (Braff 2015: 156), Dr. Catalina explained, ‘I do not think we should do anything, not donate them, or thaw them. I do not think they are from us, they are from the couple’. On the contrary, the clinic administrators were concerned about potential legal repercussions if they discarded cryopreserved embryos. Similar to Cromer's (2018) observations in the US, the lack of legislation addressing this predicament makes room for potential legal conflict between couples and the clinic.

A combination of medical staff's moral understanding of what embryos represent and fears of legal reprimand, results in the continued accumulation of extra embryos and continuous costs accrued by the clinic, a predicament affecting ART centres globally. Despite this growing concern, no protocols or multinational discussions are addressing the issue. Individual clinics (or independent embryo adoption or donation organisations as described by Cromer 2018) are left to improvise their own approach as they untangle the ethical and moral notions that surround frozen embryos.

## Discussion & conclusion

In this article I have considered doctors’ perceptions of particular ART techniques and how they negotiate the use of certain practices based on their own morals, social understandings, and the demands of running a professional private clinic. My analysis demonstrates that this context of limited legal regulations combined with the competition of private medicine impacts the praxis of assisted conception on three interconnected levels: the medical system of ARTs, doctor–patient relations and doctors’ relationship with society.

On the first level, generally speaking, the medical system of assisted conception is affected in two ways: treatment variation and competition. Limited regulation creates treatment variation across clinics and even between doctors at the same clinic as doctors choose which treatments they will utilise. This allows patients to shop around for the type of treatment they desire (Shaw 2016: 124‐127), but it may also lead to contradictory diagnoses and treatments, and over‐medicalisation (Hollos *et al*. 2009).

The nature of private medicine also drives competition between doctors, preventing collaboration between doctors as well as between doctors and other professionals working in reproductive health. This ultimately eliminates a resource doctors could access to discuss personal positions on certain techniques or deliberate cases that pose ethical concern. The high financial costs of operating a private medical facility also impacts treatment costs and who can access treatments in a medical system where ARTs are not covered by health insurance. As my findings indicate, however, the doctors’ practices were not influenced merely by financial demands and the prospect of financial gain, a concern that has been raised about the privatisation of health care (e.g. Myser 2011).

The second level involves doctor–patient interactions. Given a lack of regulations, doctors negotiate treatment boundaries based on their own morals, social values and professional obligations to patients. Despite being aware of standards set by medical associations, medical professionals used their own moral judgments and social understandings to justify treatment decisions, shedding light on how doctors envision biomedical advancements to correspond with their local moral worlds (Kleinman 1992). This was evident in doctors’ decisions to terminate treatment early when embryos were interpreted to lack the necessary quality for transfer. Such decisions were made with the best intentions for the patient in mind, although other doctors may have proceeded with treatment. Without protocols in place, fertility specialists have to judge for themselves which path they believe will be the least detrimental for the patient.

The third level involves the relationship between society and doctors’ treatment boundaries. Utilising some treatment techniques but limiting others directly impacts the types of treatments available and who has access. Ultimately, then, doctors are making decisions regarding who should be parents, how families should be formed and the fate and significance of extra embryos. These are socially and morally charged questions that shape reproductive and relational potential – in both the present and the future (Simpson 2013: S95). Lack of legislation, therefore, not only allows treatment practices to vary across clinics, but also social and moral understandings of these technologies, who should access them, and in which ways they should be accessed also vary. As suggested by Dr. Mabel's and Jessica's conflicting ideas about acceptable forms of egg donation, local values and notions of family formation are multiple. There is not only conflict between the ‘universal’ medical perspective and the vernacular (Simpson 2013), but rather multiple overlapping and competing local norms.

Although doctors may not see their role as necessarily intervening in the social and moral aspects of patients’ lives, they do so both directly and indirectly as they negotiate the boundaries of their practice. As in Livingston's (2012) account of oncology practice in Botswana in the early 2000s, where diagnostic technologies, medications and medical expertise were scarce, doctors had to improvise their medical practice – making hard decisions about whom to treat, what supplies to utilise, and ultimately who would live or die. In the context of limited regulations, collaboration, and ethical guidelines analysed here, a similar situation arises, but in regard to what can be called ‘improvised ethics’. Doctors improvise in their everyday practice due to the competition between clinics and a lack of regulatory guidelines, a context created by limited government involvement in infertility treatment. This lack of government contribution, financial and legal, is a double‐edge sword as it gives doctors the ability to practice as they see fit, but also isolates them from collaborating with others and developing a consensus on what constitutes ethical and socially appropriate practice. In *Testing Women, Testing the Fetus*, Rapp (1999) concludes that relegating women's reproductive capacities, desires, and decisions to the private realm deprives wider society of their experiences and knowledge, including other women trying to traverse the ethical and moral quandaries posed by these uncharted technologies. In a similar vein, the resources of fertility specialists – their experiences, knowledge and perceptions – are also being lost, as doctors’ practise independently. They struggle independently to make morally responsible treatment decisions based within their comprehension of these evolving technologies and their effects on society.

These findings effectively show that as biomedicine and the life sciences continue to advance, and as the technical understandings of reproductive processes continue to be further broken down into putatively more precise and objective stages of early life (Franklin 2006, Lappé and Landecker 2015), new more ambiguous and dynamic ways of attributing significance to these life stages are arising. The empirical data analysed here demonstrates that doctors interpret the significance of an embryo at a specific moment based on its perceived quality and quantity (or that of the gametes that created the embryo), kin connections, financial demands, and, ultimately, developmental potential. Although embryos are attributed innate significance as living entities, the meanings of the embryos’ divided life stages are connected to interpretations of past and future life stages and the situation that surrounds a particular embryo. There is not one life span of the embryo, but rather multiple, individual life paths and doctors are, in this context, heavily involved in determining the contours of these paths, using their own personal morals, improvised ethics and individual experiences to build them into, but also cut through, the broader social landscape. While one may imagine them to be ‘shorn of the social and the personal’, medical practices are inherently social (Lambert and McDonald 2009: 5). Therefore, when doctors navigate the techniques of assisted conception in their everyday practices, they become more than just medical or entrepreneurial pioneers. They become forces for shaping the social world.

## Notes

1 Prices based on treatment costs during fieldwork, 2012‐2013.

2 US$ 1 = COP 2800.
